# Zinc as a Gatekeeper of Immune Function

**DOI:** 10.3390/nu9121286

**Published:** 2017-11-25

**Authors:** Inga Wessels, Martina Maywald, Lothar Rink

**Affiliations:** Institute of Immunology, Faculty of Medicine, University Hospital RWTH Aachen, Pauwelsstr. 30, 52074 Aachen, Germany; iwessels@ukaachen.de (I.W.); mmaywald@ukaachen.de (M.M.)

**Keywords:** zinc flux, zinc wave, homeostatic zinc signal, immune function, zinc deficiency, signaling pathways

## Abstract

After the discovery of zinc deficiency in the 1960s, it soon became clear that zinc is essential for the function of the immune system. Zinc ions are involved in regulating intracellular signaling pathways in innate and adaptive immune cells. Zinc homeostasis is largely controlled via the expression and action of zinc “importers” (ZIP 1–14), zinc “exporters” (ZnT 1–10), and zinc-binding proteins. Anti-inflammatory and anti-oxidant properties of zinc have long been documented, however, underlying mechanisms are still not entirely clear. Here, we report molecular mechanisms underlying the development of a pro-inflammatory phenotype during zinc deficiency. Furthermore, we describe links between altered zinc homeostasis and disease development. Consequently, the benefits of zinc supplementation for a malfunctioning immune system become clear. This article will focus on underlying mechanisms responsible for the regulation of cellular signaling by alterations in zinc homeostasis. Effects of fast zinc flux, intermediate “zinc waves”, and late homeostatic zinc signals will be discriminated. Description of zinc homeostasis-related effects on the activation of key signaling molecules, as well as on epigenetic modifications, are included to emphasize the role of zinc as a gatekeeper of immune function.

## 1. Introduction

The transition metal zinc has long been known to be essential for the growth of fungi and development in rodents [1,2]. Symptoms arising from zinc-deficiency in animal studies were reported to be growth failure, loss of hair, testicular atrophy, as well as thickening and hyperkeratinization of the epidermis. Despite these observations, it took a century until zinc was accepted to be essential for human beings [3]. 

Previously, zinc deficiency was considered to be rare. Nowadays, however, zinc deficiency is known to be very common, especially in developing countries [4]. Worldwide, about two billion people are estimated to be affected by zinc deficiency. In developing countries, zinc deficiency is the 5th leading cause for the loss of healthy life years. In industrial countries, mainly the elderly population is affected by zinc deficiency [5]. Nearly 30% of the elderly population is considered to be zinc deficient. Since zinc homeostasis is known to be important in immunological reactions such as the inflammatory response, and the oxidative stress response, multiple chronic diseases observed in the elderly are probably related to zinc deficiency. Hence, diseases such as Rheumatoid Arthritis, diabetes, atherosclerosis, impaired cognitive function, as well as age-related macular degeneration (AMD) may be due to zinc deficiency, worsening chronic inflammation and triggering oxidative stress [6,7,8,9]. Besides the elderly, vegetarians or vegans and patients suffering from renal insufficiency or chronic diarrhea are affected by zinc deficiency [6,9,10,11,12]. In humans, clinical manifestations of zinc deficiency are similar to those observed in rodents and include weight loss, growth retardation, atrophy, and immune dysfunction, as well as increased oxidative stress and a boosted inflammatory immune response [3,13,14,15,16]. Dietary insufficiency and/or compromised uptake can result in perturbation of zinc homeostasis in humans [12,17,18]. 

Besides zinc, vitamins and other transition metals such as copper and iron, as well as calcium are essential for adequate immune function. Deficiency or exceeding levels of those nutrients can be associated with the occurrence of distinct diseases. In this regard, a multitude of diseases such as Rheumatoid Arthritis [19], multiple sclerosis [20,21,22] impaired cognitive function as seen in Alzheimer’s disease [7,23], and AMD [24] were reported to go along with skewed concentrations of the above-mentioned nutrients.

## 2. Zinc Metabolism and Homeostasis

At 2–3 g in total, zinc is the second most abundant metal in humans and is distributed unequally throughout different organs and tissues. Prostate, pancreas, and bone are considerably high in zinc, containing up to 200 µg/g. In contrast, zinc concentrations in heart, brain and plasma are comparatively low at 1–23 µg/g. Although plasma has only 1 µg/g, it is probably the most important reservoir for zinc homeostasis [5]. 

Red meat and oysters are rich dietary sources of zinc [5]. Foods prepared from unrefined cereals, legumes, or plant parts rich in phytates decrease zinc bioavailability, by binding zinc very effectively [25]. Zinc is resorbed in the intestine by specific zinc transporting proteins and distributed in the human body. In serum, free zinc is rare since it is largely bound to proteins such as albumin, α_2_-macroglobulin (A2M), and transferrin. Albumin binds zinc with a relatively low affinity, A2M with a medium affinity and transferrin with a high affinity. Subcellularly, zinc is distributed between specific zinc-storing vesicles called zincosomes (~50%), the nucleus (~30–40%), and the remainder is distributed between the cytoplasm and other organelles [26,27]. In the cytoplasm, zinc is largely bound by zinc-chelating proteins called metallothioneins (MTs) discovered in 1957 [28]. MTs play an important role in zinc homeostasis by complexing about 20% of intracellular zinc. MT-1 for example binds up to seven zinc ions, making it specifically suitable to function as a zinc buffer [29]. During evolution, cells not only evolved metal-binding proteins to maintain low cytosolic metal ion concentrations to protect themselves against high cytotoxic metal concentrations, but also mechanisms of zinc compartmentalization and zinc sequestration. Modulation of available cytosolic free zinc is facilitated by zinc storage in zincosomes or other organelles such as endoplasmic reticulum (ER) or Golgi apparatus respectively [30]. Zinc distribution between the cytosol an organelles is mediated by specific zinc transporting proteins, such as zinc importers or zinc exporters, or via membrane channels [29,31]. The cytosolic free zinc ion concentration is maintained at remarkably low picomolar to nanomolar concentrations [32]. However, through zinc release from protein and organelles zinc concentrations can increase transiently and locally, enabling zinc to affect gene expression, enzymatic activity, and cell signaling [31,33]. 

Zinc homeostasis is particularly controlled by 24 zinc transporting proteins, and four MTs (MT1-4), playing coordinated roles in the distribution, transport, and maintenance of intracellular zinc level. MT-1 and MT-2 are expressed ubiquitously throughout the human body, play an important role in liver and kidney function and are inducible by various stimuli such as metal ions, cytokines, glucocorticoids, and oxidative stress, respectively. In contrast, MT-3 and MT-4 are expressed cell specific. MT-3 is primarily expressed in the central nervous system, whereas MT-4 is primarily found in epithelial tissue. The expression of both MT3 and MT4 is very strictly controlled, highlighting distinct functions of MT isoforms in different organs and tissues [34,35,36,37].

Based on their membrane topology, zinc transporters are divided into two major families: (1) 14 zinc transporters of the SLC39s family (Zrt (zinc-regulated transporter)-like, Irt (iron-regulated transporter)-like proteins, ZIP 1–14) that increase cytoplasmic zinc and (2) 10 zinc transporters (ZnT 1–10) of the SLC30s family that lower cytoplasmic zinc by transporting zinc either out of the cell or into intracellular organelles [38]. Since zinc transporters are found in a myriad of different cell types in the human body, both on the outer plasma membrane, and on organelle-membranes, such as mitochondria, Golgi apparatus, lysosomes, and ER, the necessity of a proper zinc level for a specific cellular function is emphasized [38,39]. Moreover, zinc distribution was more recently mentioned in relation to receptors and ion channels other than ZIPs or ZnT, for example voltage-dependent calcium channels, transient receptor potential channels, nicotinic acetylcholine receptors, and glutamatergic receptors. Additionally, diffusion of zinc bound to amino acids is known. However, these mechanisms need to be investigated more in-depth [39]. 

Interestingly, zinc-dependence was found in all classes of enzymes, i.e., transferases, hydrolases, lyases, isomerases, oxidoreductases, and ligases [40,41]. The physiological importance of zinc was moreover supported by in silico studies which showed that about 10% of the overall human proteome can potentially bind zinc. Zinc binding can be facilitated by a variety of binding motifs including: (1) zinc-finger motifs; (2) Really Interesting New Gene (RING) finger domains; (3) LIM domains composed of two adjacent zinc finger domains; and (4) plant homeodomain (PHD) domains similar to RING fingers [42]. Thus, interaction of the transition metal zinc with proteins is highly complex. 

The zinc proteome consists of two major groups, comprising enzymes and transcription factors respectively. Those cover 90% of the overall zinc proteome, indicating that zinc is involved in regulating catalysis and transcription [43,44]. Because zinc is required for catalysis by some enzymes, as well as for maintaining enzyme structure [26], intracellular signaling pathways can be altered due to altered zinc availability, affecting cellular maturation, differentiation, and function [45].

## 3. Immune Function during Zinc Deficiency

The importance of zinc for proper immune function is best observed in zinc-deficient individuals. Zinc deficiency has been known for 50 years [15] and is associated with skin abnormalities, hypogonadism, cognitive impairment, growth retardation, and imbalanced immune reactions which favor allergies and autoimmune diseases [3,5]. In the case of inherited malfunction of zinc homeostasis, as seen in Acrodermatitis Enteropathica, zinc deficiency can be lethal [17]. 

Zinc deficiency can be classified by severity and is divided into severe or marginal zinc deficiency, respectively. Severe zinc deficiency is often observed because of malfunction of zinc uptake in the intestine. This is reported in patients suffering from chronic diarrhea, patients being treated with penicillamine, patients receiving parenteral nutrition without zinc, or following excessive alcohol consumption [46,47,48,49]. Patients present clinical symptoms such as lymphopenia, decreased ratios of T helper (Th) cells to cytotoxic T cells, decreased natural killer (NK) cell activity, and increased monocyte cytotoxicity. The most severe form of zinc deficiency observed in Acrodermatitis Enteropathica. This zinc malabsorption syndrome is inherited as an autosomal recessive condition and is due to a mutation of the intestinal zinc uptake protein ZIP4 [17,50]. Acrodermatitis Enteropathica is characterized mainly by diarrhea, weight loss, recurrent viral and bacterial infections, dermatitis, hair loss, and neuropsychological disturbances [51]. However, all observed symptoms can be corrected by high dose (1 mg/kg) zinc supplementation [52]. Other zinc transporter-related diseases are discussed later in detail.

Marginal zinc deficiency is characterized by slight weight loss, rough skin, oligospermia, and hyperammonemia [47]. It is probably caused by nutritional zinc deficiency, often seen in vegetarians or vegans, due to the consumption of high levels of zinc-chelating agents in food originating from cereals, legumes, or plant parts. In these foods, lignin and phytates counteract zinc absorption by binding zinc and reducing its bioavailability [10,53]. Nutritionally related marginal zinc deficiency is prevalent in the elderly population. Hence, a correlation between impaired immune function and zinc status is likely in older people [6,54,55]. Within the seventh decade of life, the human immune system undergoes dramatic age-related changes, termed “immunosenescence”. Associated with this condition is an increased incidence of inflammatory disease, most notably cardiovascular diseases, whereas the immunological response to vaccines is typically impaired [56]. The underlying zinc-modulated molecular mechanisms and signaling pathways are discussed below.

The overall frequency of zinc deficiency worldwide is estimated to be higher than 20% [18]. Interestingly, zinc supplementation is already widely practiced and approved for clinical treatment of multiple diseases. Zinc has proven to be very effective for the treatment of pediatric diarrhea, saving millions of children’s lives in developing countries such as India [48]. The Food and Drug Administration (FDA) approved zinc supplementation for the treatment of Wilson’s disease, a genetic disorder in which copper builds up in the human body [57]. In the elderly population, age-related macular degeneration (AMD) is of frequent occurrence and AMD progress, which can result in blindness, can be treated successfully by zinc supplementation [58,59]. Furthermore, not only the very young or elderly benefit from zinc supplementation, as shown by studies with patients suffering from: (1) viral infections, e.g., the common cold, diarrhea, chronic hepatitis C, or human immunodeficiency virus (HIV); (2) bacterial infections such as *shigellosis* or *Helicobacter pylori*; (3) parasitic infestations such as acute cutaneous leishmaniosis or malaria; (4) autoimmune diseases such as Type 1 Diabetes Mellitus (T1DM) and Rheumatoid Arthritis; and (5) transplant rejections [60]. This widespread variety of clinical manifestations makes zinc deficiency a serious nutritional problem. However, to date, no reliable biomarker to assess zinc status exists [61]. Thus, zinc deficiency is difficult to diagnose. Overall, zinc contributes to the overall regulation of immune cell function, influencing several signaling pathways. Hereby, zinc acts in a direct manner by binding reversibly to regulatory sites in signaling proteins, or indirectly by influencing enzymes such as phosphatases which are a component of and regulate signaling pathways [45]. 

Zinc homeostasis is essential for multiple aspects of the immune system including hematopoiesis, cell maturation and cell differentiation, cell cycle progression, and for the proper function of immune-cells [62]. During inflammation, adequate zinc status is essential since in acute phase responses zinc is transiently transferred from serum into organs, especially the liver, causing transient serum hypozincemia. This transient loss of serum zinc is eventually rebalanced during resolution of the inflammatory response. Here, zinc is probably released from tissue into serum. One proposed reason for this complex mechanism is to act as a danger signal for immune cells [63]. Since extracellular microorganisms are also dependent on zinc availability, zinc sequestration by the human immune system helps to combat invading pathogens. This is facilitated due to expression of pro-inflammatory acute phase proteins including interleukin (IL)-6, which upregulates expression of zinc binding peptides such as MTs and A2M [64]. In immune cells, on the one hand, increased intracellular zinc levels can intoxicate engulfed pathogens and act cytoprotectively by neutralizing reactive oxygen species (ROS) and nitrogen species (RNS). In general, zinc homeostasis and zinc signals are crucial to counteract inflammatory diseases, and the correlation of undernourishment with severe inflammatory diseases is accompanied by prolonged and severe forms of serum hypozincemia. In the literature, it has been suggested that hypozincemia goes along with elevated inflammatory mediators, e.g., ROS, and antimicrobial peptides such as calprotectin or matrix metalloproteases (MMP), causing tissue injury, especially in liver, lung, and spleen [63,65,66].

In general, cellular function, such as the intracellular killing of harmful pathogens, cytokine production as well as ROS production, are dependent on zinc and are impaired due to zinc deficiency. Zinc deficiency also adversely affects the maturation and function of T and B cells, which occurs through dysregulation of basic biological functions at the cellular level, described later in this review in more detail. For T cells, a disturbed ratio of Th1 and Th2 cells in favor of Th2-driven allergic reactions is a well-known consequence of zinc deficiency [67]. Zinc flux and homeostatic zinc signals, as defined below, are highly important for adequate T cell differentiation, and this observed malfunction can be reversed by zinc supplementation [68,69,70,71]. Moreover, the pro-tolerogenic immunoreaction is triggered by long-lasting changes in intracellular zinc levels due to induction of regulatory T cells (Treg) cells and dampening of pro-inflammatory Th17 and Th9 cells [72,73,74,75]. 

T cell development is strongly dependent on DC activation. Interestingly, zinc signals were recently shown to induce a tolerogenic DC phenotype in vitro and in vivo. Herein, zinc suppressed MHC-II expression and enhanced programmed cell death 1 ligand 1 (PD-L1) and PD-L2 expression resulting in the manipulation of the Treg/Th17 balance in favor of Treg cell development [76]. Moreover, on the molecular level, zinc inhibits the IL-6-induced STAT3 signaling cascade essential for Th17 development [74]. Another potential target of zinc-mediated Th17 manipulation might be found on the epigenetic level since several epigenetic enzymes as (de-)acetylases, and (de-)methylases are regulated in a zinc dependent manner [72,77]. 

A malfunctioning adaptive immune system has been observed in the elderly population, whereby secretion of pro-inflammatory IL-6 is pathologically elevated, while T cell activation was reduced, as were responses to stimulation or vaccination [6,56,68,70,78,79]. Interestingly, all these pathologies can be improved due to zinc supplementation, highlighting the significance of zinc for a well-balanced immunoreaction.

Regarding innate immunity, zinc deficiency leads to prioritization of maturation of innate immune cells such as monocytes. In this case, differentiation was promoted by the reduction in the concentration of intracellular free zinc [80]. This was facilitated by induced expression of the zinc-binding heterodimeric protein calprotectin [81]. In general, calprotectin was also highly expressed in neutrophils [81,82]. Recently, zinc deficiency has been shown to negatively influence critical neutrophil functions such as phagocytosis, oxidative burst, degranulation, cytokine production, chemotaxis, and neutrophil extracellular trap (NET) formation [83,84]. These observations are in line with an impaired ROS production during zinc deficiency, which is needed for NET formation, and for intracellular killing of phagocytosed pathogens by neutrophils [85,86]. 

Furthermore, NK cell function was weakened when zinc signals are absent. In this regard, recognition of major histocompatibility complex class I (MHC-I) on target cells, as well as lytic activity, was decreased [87,88,89,90]. In contrast, zinc supplementation increased differentiation of CD341 progenitors toward NK cells and their cytotoxic activity [91], as well as NK killing activity, and intracellular perforin concentrations [90].

Besides cellular immune responses, zinc is also indispensable for proper RNA transcription, DNA synthesis, as well as cell survival [27,92]. In relation to cell survival and apoptosis, adequate intracellular zinc levels are needed because apoptosis is triggered by zinc deficiency. Furthermore, cytokine function and secretion are adversely affected by zinc deficiency impairing the function of the basic messengers of the immune system. Thus, zinc is crucial for the appropriate development and function of the whole immune system including innate as well as adaptive immunity, and the affected signaling cascades and networks are described in detail below.

## 4. Types of Zinc Signals

As indicated earlier, intracellular as well as extracellular zinc is usually bound to protein. From protein, zinc can readily be made available but also made unavailable. About 10% of total human proteins bind some zinc throughout the lifetime of the protein, as it is essential for catalytic or structural functions. In these cases, zinc is not released under normal circumstances. Other proteins revealed rapid association and dissociation rates for zinc, short enough for zinc to regulate processes such as pathogen sensing, intracellular transportation and signaling. This pool of zinc is called “free”, “labile” or “mobile” zinc, describing loosely bound or unbound ions that are thermodynamically available for cellular metabolism. Coordination chemistry of those “mobile” zinc ions has been barely explored [30]. Thus, it is difficult to create artificial substances that can deliver zinc or chelate it away from protein. This explains why there is so far no clear answer to the question, which zinc compounds are best to use for zinc supplementation. In addition, it underlines why there is still no reliable method to measure zinc status of individuals and the immense discrepancies between studies even if experimental set ups seem to only differ slightly. 

Zinc is released from bound protein during regular protein turnover. It is mobilized from protein by chemical processes, and can be mobilized between the extracellular and intracellular, organellar or vesicular compartments [30]. Efficient zinc buffering inside the cells, to avoid zinc-intoxication and zinc-deficiency, is regulated by coordinating expression of zinc binding proteins and zinc transporters [37]. This includes so-called muffling, where proteins transiently bind zinc and transport it to vesicles [29]. To be correct, zinc ions are never completely “free” inside the cell but always complexed with amino acids, phosphates or other low molecular weight ligands. However, “free zinc” will be used here, to distinguish the pool of zinc that is available for signaling from zinc stably bound to protein and therefore unavailable. This is the term, generally used in literature in this field, although it is not a satisfactory definition in the chemical sense [30].

After stimulation of cells, intracellular zinc fluctuation, or in short “zinc flux”, has been observed, suggesting a function for zinc as a second messenger [93,94,95]. In myeloid cell lines, primary monocytes and granulocytes, a zinc flux can be induced by lipopolysaccharide (LPS) or phorbol-12-myristate-13-acetate (PMA), which was not detected in lymphoid cells [83,94]. Furthermore, Fc-epsilon receptor I (FcεRI) activation caused a zinc flux in mast cells [96]. Increased intracellular zinc in monocytes was also detected after stimulation with monocyte chemoattractant protein (MCP-1), tumor necrosis factor (TNF)α, insulin and Pam3CSK4 [97]. Using various ligands for toll like receptors (TLR) including Pam3CSK4 (TLR1/2), *Listeria monocytogenes* (TLR2), flagellin (TLR5), FSL-1 (TLR6/2), ssRNA40 (TLR7) and inhibitory oligonucleotides (ODN) 1826 (TLR9) all increased intracellular zinc in murine macrophages and primary human monocytes [94,97]. In these cases, zinc was mostly shown to be increased, but a decrease might occur as well. Zinc can transduce the extracellular stimulus into an intracellular signaling event. Release of zinc from the endoplasmic reticulum has been shown to be inducible by some hormones, similarly to what has been described for calcium [98]. Another source of zinc is zinc-binding-proteins as already indicated. Here, MTs play a decisive role, as they bind up to seven zinc ions, which can be released rapidly. Zinc ions can be released from their coordination environment with sulfur donors. Zinc is released from cysteine in proteins, suggesting that a redox signal can be “translated” into a zinc signal [99], which we will return to later in this review.

**Figure 1:**
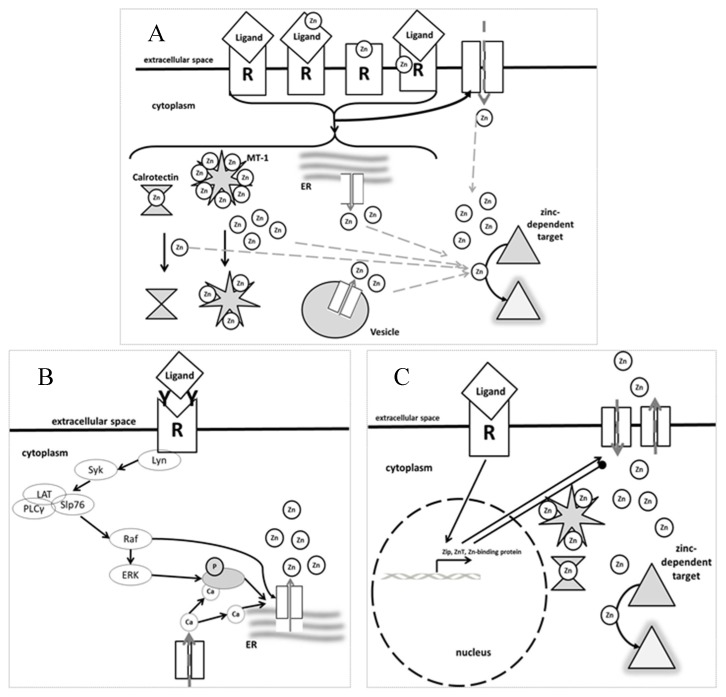
Different Types of Zinc Signals: (**A**) Zinc Flux, as observed after receptor triggering (e.g., binding of lipopolysaccharide (LPS) to Toll like receptor (TLR)4), is generated within seconds. (**B**) A Zinc Wave, as is induced via immunoglobulin receptors and involving calcium flux, can be observed within a few minutes. (**C**) Homeostatic Zinc Signals, for example as observed after LPS stimulation of dendritic cells, take a few hours to be established and involve the expression of zinc transport and binding proteins. For explanations see the text. Abbreviations: ER: endoplasmic reticulum; ERK: extracellular signal-regulated kinase; MT: metallothionein; PLC: phospholipase c; R: receptor; Slp76, SH2 domain-containing leukocyte protein, 76 kD. Modified after [62,96,105].

As the zinc flux occurs within seconds to minutes of stimulation, it is not due to changes in gene expression, but alteration of activity of existing agents. Figure 1 illustrates that not only fast zinc fluxes exist, but also a so called “zinc wave”, which occurs within a few minutes. For the zinc wave, the influx of calcium is essential. This has been described after cross-linking of FcεRI in mast cells [96]. Furthermore, a delayed signal occurs a few hours after stimulation. Regarding this zinc signal, a certain stimulus activates expression of genes involved in zinc metabolism, including zinc transporters and zinc binding proteins, causing alteration of intracellular zinc levels some time after the initial stimulus. This third type of zinc signal is said to have mostly homeostatic functions and will therefore be named accordingly here. Here, intracellular zinc levels are changed long-term, i.e., permanently elevated or decreased compared to the original concentration measured inside the cell before stimulation occurred. The homeostatic zinc signal was shown to be important for major cellular changes such as the process of maturation and differentiation of myeloid and dendritic cells [80,100]. In B and T cells, stimulation induces a sustained increase in intracellular zinc due to downregulation of ZnT1, ZnT 4–7 and upregulation of ZIP6, ZIP8, and ZIP10 [60,101]. When ZIP6 and ZIP8 were silenced, cytokine production and proliferation of T cells was blocked [71,101,102]. Similarly, BCR-induced signaling was disrupted in cells from ZIP10 knockout mice [103]. Various activation signals, including mediators of diseases, change the expression of MTs, enabling regulation of zinc homeostasis in the long term as well [80,104].

## 5. Effects of Zinc in Immune Cell Signaling

Changes in extracellular zinc levels, such as serum hypozincemia during acute phase reactions, have been suggested to activate immune cells, functioning as a “danger signal”. In addition, cytokines, integrin binding, growth factors and other immune cell receptor ligands trigger intracellular zinc flux. In recent years more and more regulatory pathways have been demonstrated in various immune cells to directly or indirectly involve zinc signaling. The following section provides a summary of recent developments, concentrating on the major mechanisms in immune cells, and for further information the reader is referred to the extensive literature on this topic [39,62,103,106]. Exemplarily, we will describe briefly key signaling pathways for cells from the adaptive as well as the innate immune system. General concepts, such as the effect of intracellular zinc concentrations on the activities of phosphodiesterases (PDE), phospho tyrosine phosphatases (PTP) and their antagonists the tyrosine kinases (TK), or the translocation of signaling molecules and transcription factors such as NFκB to the nucleus, can probably be extrapolated to other examples of receptor-induced signaling pathways. However, cell type dependent exceptions to the paradigm might exist, underlining the importance of testing the general concepts for each individual cell type and pathway. 

The ideal immune response should be fast and specific against each kind of molecular pattern associated with extracellular or intracellular pathogens, and degenerate or damaged cells. Instead of assigning all the necessary functions to one single cell type, the immune response is optimized by the cooperation of various cell types, supported by soluble mediators with distinct functions, roughly separated into innate, fast responding cells and highly specific adaptive immune cells. Quiescent cells circulate in the bloodstream and enter the tissues after activation in case of an infection. Here, firstly innate immune cells, composing mainly granulocytes and monocytes, but also NK cells, mast cells and dendritic cells are recruited. These cells can recognize general pathogen-associated molecular patterns (PAMPs) via surface receptors denoted as pattern recognition receptors (PRRs). Examples of PRRs include the Toll-like receptors (TLRs), with at least 10 members in humans, nucleotide-binding oligomerization domain-like receptors (NLRs), and retinoic acid-inducible gene-I-like receptors (RLRs). Receptor triggering activates signaling pathways and subsequent anti-microbial activities such as degranulation, phagocytosis and pathogen-killing, the presentation of the antigen to other (adaptive immune) cells and cytokine production [107]. Adaptive immune cells, resembling T- and B-lymphocytes, are in contrast highly specific, but require a longer time to become fully active. Each cell has a receptor, called B cell receptor (BCR) or T cell receptor (TCR), and thereby specificity for a certain antigen [108,109]. Triggering of the naïve cell by its matching antigen activates intracellular signaling and causes the proliferation of this particular cell clone and its differentiation into an effector or memory cell. Binding of co-stimulatory molecules, for example interleukins or interferons, to their receptors is necessary for the fine-tuning of the pathogen-specific response. Adaptive immune cells carrying receptors for autoantigens, are selected and silenced. 

### 5.1. Zinc Signals Can Regulate Phosphatase and Kinase Activities

The translation of an initial stimulus, detected by a cell surface receptor, in to gene expression in the nucleus is largely mediated by transferring phosphate residues from one signaling molecule to the next in a consecutive cascade [110]. Here, tyrosine, serine and threonine residues in signaling proteins are the acceptors of the phosphate residues in most cases. Phosphorylation is mediated by protein kinases, while protein phosphatases are responsible for removing phosphates. Combining results for tyrosine phosphorylation of multiple studies, one can assume that phosphorylation is balanced by zinc levels, as zinc affects the decision over the activity of protein tyrosine kinases (PTKs) and phosphatase (PTPs). Phosphorylation of a target protein can result in an activation or an inactivation of that protein and each case must be specifically examined [111].

Several PTKs, belonging largely to the Src family, were shown to act in a zinc–dependent manner. Here, zinc supported the phosphorylation of target molecules. Examples include p60c-Src as found in Alzheimer’s patients, anaplastic lymphoma kinase, which is a member of the receptor tyrosine family, Bruton’s kinase (BTK) and also mitochondrial Src tyrosine kinases, showing that kinases from various cellular compartments can be affected by zinc levels [112,113,114,115,116]. 

A group of protein kinases important for signaling in immune cells is the protein kinase C (PKC) family, reported to be zinc metallo-enzymes. These are serine/threonine kinases of several isoforms, including classic PKC, which are activated by cofactors including diacylglycerol (DAG) or calcium, novel PKCs that do not bind calcium but only DAG and atypical PKCs which bind neither of these co-factors. Zinc was reported to be important for PKC’s structure. Approximately four zinc ions were found associated with each PKCα subunit and similar values have been reported for PKCβII and PKCγ. This matches well with PKC’s C1 domain forming four Cys3His binding motifs, probably occupied by zinc. As this domain is conserved across all PKC isoforms, zinc might be structurally important for all PKCs [117]. Whether more zinc ions can be bound is questionable, but a release of zinc from PKC after incubation with lipid second messengers or due to thiol oxidation has been noted [118]. Activation of PKC, especially by homeostatic zinc signals, has been shown. Thus, amongst others, binding of phorbol esters to the cytoskeleton and the plasma membrane has been found to require zinc [119]. IKK and NFκB activity was decreased in macrophages negative for PKCε. LPS stimulation of such PKCε knockout macrophages, showed lower ERK and p38 phosphorylation compared to wildtype controls, suggesting that here, the zinc signal alters signal transduction via PKC [120].

In this context, the tight interrelation of zinc homeostasis with redox metabolism should be mentioned. PKC, activated through PMA, subsequently causes the production of reactive oxygen species by nicotinamide adenine dinucleotide phosphate (NADPH) oxidase. Induction of a zinc flux by PMA was mentioned earlier [93]. When NADPH oxidase was inhibited, no increase in intracellular zinc was observed after PMA stimulation [66]. Incubation of cells with H_2_O_2_ on the other hand, elicited elevated intracellular zinc concentrations, suggesting an association of ROS production with the occurrence of a zinc signal. In this case, the source of zinc might be PKC itself, or zinc-binding proteins such as MT, both known to release zinc when oxidized. Alternatively, zinc might be released from cellular compartments in an oxidant-sensitive way [71,94,101,121,122]. Maybe as a feedback mechanism, zinc affects ROS production as well. Incubation of cells with a high concentration (100 µM) of the zinc chelator TPEN caused altered mobilization of PKC between plasma membrane and the cytoskeleton and inhibition of PKC activity [93,123,124]. When myeloid cells were depleted of zinc by incubation in zinc-deficient medium, basal and stimulation-induced ROS levels were increased [66]. Increased ROS production in zinc deficient neutrophils was also observed [66,84,125,126,127]. ROS as well as zinc are essential second messengers for the activation of various functions in neutrophils and other immune cells [83].

Activity of PKA and PKG (both serin/threonine kinases) are mainly affected by levels of the second messengers cyclic adenosine monophosphate (cAMP) and cyclic guanosine monophosphate (cGMP), respectively. Increased cGMP additionally cross-activates PKA. PDEs are zinc dependent and hydrolysis of both cAMP and cGMP has been shown to be affected by zinc. Zinc is tightly bound to the catalytic center of PDE, but PDE also has an inhibitory site, where available zinc binds if present in elevated concentrations [128,129]. Furthermore, it was shown that zinc affects LPS-induced expression of certain PDEs, including PDE4B [130]. Therefore, cAMP and cGMP levels increased if high zinc concentrations were available because hydrolysis by PDE was inhibited. This was shown for PDE 4, 3, and 1, which were all inhibited by zinc in monocytes. Zinc flux activated adenylate cyclase (AC) while the enzyme was inhibited by homeostatic zinc signals, probably as a feedback mechanism to resolve stimulation-mediated effects. Alterations in guanylate cyclase (GC) activity were only affected by the zinc flux [131,132]. Downstream targets of PKA include Raf-1, responsible for NFκB activation, while PKG targets members of the MAPK family, offering another mechanism of zinc’s effect on signaling, which remains to be investigated in more detail [133]. 

As suggested earlier, zinc seems to balance phosphorylation of signaling molecules in direct and indirect ways, altering the activities of kinases and their antagonists, the phosphatases. PTPs are largely regulated by proteolysis, dimerization, the redox state of the cell and phosphorylation and dephosphorylation. Zinc is one more regulator to add to the list, as was shown amongst others for PTP1B (IC_50_ (zinc) = 3–17 nM), SHP-1 (IC_50_ (zinc) = 93 nM, SHP-2 (IC_50_ (zinc) = 1–2 µM), PTPRB (IC_50_ (zinc) = 98 pM), PTEN (IC_50_ (zinc) = 0.6 nM) [134,135] and the same might be true for several others from the list of 107 PTPs in humans. IC_50_ values all fit well with the physiological concentration of zinc within cells, so that regulation of their activity by zinc signals is plausible. Suggested mechanism underlying the regulation of PTP activity by zinc include the displacement of co-factors by zinc and the direct interaction of zinc with the catalytic center of PTPs [136]. Prolonged protein tyrosine phosphorylation was described in many signaling pathways of immune cells [94,97,137]. An effect of zinc has thus not been investigated in all of them, but its involvement in regulating those signaling pathways is likely. Similar inhibitory effects of zinc were found for MAPK phosphatases (MKP), which are closely related to PTPs, but largely affected the phosphorylation of threonine residues [138] and for calcineurin (CN), a threonine/serine phosphatase (IC_50_ (zinc) = 10 nM–10 µM). Because the range of zinc IC_50_ for this enzyme is large, the effect of an intracellular zinc signal might be questionable. However, in vitro investigations in T cells revealed that CN’s activity was indeed decreased by zinc. In this case, replacement of catalytically active nickel by zinc was found as one underlying mechanism [101,139,140]. 

Regarding intracellular signaling, the major targets of protein kinases and protein phosphatases are members of the mitogen activated protein kinase (MAPK) family. Here, phosphorylation of extracellular Signal-regulated Kinase (ERK), MAPK/Erk kinase (MEK), p38 has been shown to vary depending on the zinc status of a cell [134,136,141,142,143]. Long-term zinc deficiency was associated with increased p38 activity in myeloid cells [66]. In contrast, zinc activated ERK in fibroblasts [144]. Differences in effect are most probably due to divergent experimental conditions and cell types, such as duration and intensity of zinc supplementation or deficiency. Another group of signaling molecules whose phosphorylation status and activity is altered by zinc are STAT molecules, as was demonstrated for STAT1, STAT3, and STAT6 [145,146]. Investigations of the role of zinc signals in the regulation of STAT5 activity showed varying results and may depend on the signaling pathway [71,135,147]. 

### 5.2. Effects of Zinc Finger Proteins on Signal Transduction

When it comes to homeostatic zinc signals, the zinc-finger protein A20 is of interest, being significantly involved in the resolution of infections. Induction of A20 expression by zinc signals, which resulted in increased intracellular A20 protein levels, was observed in pre-monocytic and endothelial cells as well as in other immune cells. In TLR- and TNFR-triggered signaling pathways, A20 was shown to down-regulate signaling, thereby ameliorating inflammation. TNF receptor associated factor (TRAF) 6 is de-ubiquinated by A20, abrogating TLR-induced signaling. A20 can remove ubiquitin from receptor interacting protein (RIP)-1. RIP-1 binding to IKKγ is thereby disturbed and NFκB is retained in the cytoplasm, also inhibiting TNFR-induced signaling [45,148,149]. Additionally, decreased signaling, induced via other receptors, was found to involve the inhibition of NFκB by A20. In line with the anti-inflammatory role of A20, expression of IL-1β, TNFα, C-reactive protein (CRP), and other inflammatory mediators, as well as lipid peroxidation, were downregulated as found in humans, mice and chicken due to altered zinc homeostasis [148,150,151,152,153].

Another zinc-finger protein which plays a role in homeostatic zinc signaling is peroxisome proliferator-activated receptor (PPAR)α. Similar to A20, PPARα expression was induced by zinc. PPARα alters the binding of NFκB to DNA and thereby abrogates the induction of pro-inflammatory cytokines and adhesion molecules [150].

### 5.3. Zinc Alters Hematopoiesis by Altering Intracellular Signaling

In mice, acute phase reaction was paralleled by transient serum hypozincemia and an increase in the leukocyte count in the blood [63]. Moreover, long-term dietary-induced zinc deficiency affected hematopoiesis in mice, causing a shift in the ratio of leukocyte subsets towards innate myeloid immune cells [154]. In humans, T cells were clearly diminished during zinc deficiency. A prioritizing of cells from the myeloid lineage (monocytes and granulocytes), at the cost of lymphoid cells (especially the T cell compartment), if zinc supply is limited, has been hypothesized in humans as well, however this remains to be proven. 

Decreased intracellular zinc levels were shown to be necessary for the differentiation of monocytes and dendritic cells and were suggested to play a role in granulopoiesis [80,100]. In dendritic cells, LPS stimulation caused expression of ZIP6 and ZIP10 as well as of ZnT-1, ZnT-4, and ZnT-6 via pathways involving TRIF, resulting in an altered cellular zinc homeostasis [100]. Alteration in TRIF signaling by the zinc status of a cell has also been described for LPS stimulated macrophages [97]. 

One centrally important molecule regulating differentiation in various cell types is STAT3. Recent studies revealed that phosphorylation, and thereby activation of this signaling molecule, was altered depending on the zinc status of a cell, while total protein concentration remained unaltered [155]. In myeloid cells, TLR3 and NLPR3 inflammasome-triggering activated the janus kinase (JAK)-STAT3 as well as the MAPK pathway. Pre-existing zinc deficiency augmented the effect of IL-6-or IL-1-induced JAK-STAT3 signaling. When zinc deficient cells were reconstituted with zinc prior to stimulation, signaling was normalized [142]. STAT3 is phosphorylated by SHP1/2, known to be zinc-dependent PTP [156]. Zinc chloride increased transient STAT3 Tyr705 phosphorylation in murine hematopoietic stem cells, maintaining pluripotency and self-renewal of the stem cells, even replacing leukemia inhibitory factor (LIF), which is normally essential for this process. After zinc incubation, expression of pluripotency genes was increased and expression of differentiation-associated genes was inhibited. Zinc was able to prevent retinoic acid-induced differentiation, at least for a restricted time-frame [143].

Zinc deficiency caused B cell proliferation via increased STAT3 phosphorylation [145]. However, increased STAT3 phosphorylation was noted when murine embryonic stem cells were incubated with zinc chloride, suggesting that cell type specific effects exist [157]. In addition, the timing was different between the studies, which might help to explain the differences. In a very recent study, zinc was shown to alter STAT3 phosphorylation in neuronal cells via redox-mediated mechanisms. Interestingly, phosphorylation patterns of STAT3 residues were different when nuclear and cytoplasmic fractions were compared [155]. Whether this effect can be extrapolated from neuronal cells to immune cells remains to be tested. However, redox status and zinc metabolism are highly intertwined, so that a similar mechanism is highly likely. Finally, STAT3 was also suggested to be decisive for the switch from regular to demand-adapted emergency myelopoiesis during inflammatory diseases such as sepsis [158]. However, the association between disease-related serum hypozincemia, STAT3 activity and emergency differentiation needs to be proven. 

Maturation and differentiation of T cells in the thymus is affected greatly by zinc availability. It has long been shown that the activity of thymulin, an essential factor for T cell development, is severely disturbed during zinc deficiency. This results in disturbed suppressor function, allogenic cytotoxicity and IL-2 production [159]. In addition, zinc deficiency seems to decrease the expression of anti-apoptotic factors such as B cell lymphoma (Bcl)-2 and Bcl-xL by T cells. In connection with the increased levels of pro-apoptotic factors detected in serum during zinc deficiency, this explains the severe reduction of T cell numbers [160]. Furthermore, zinc was shown to affect caspase activity. The cell cycle regulator p21 was cleaved by caspase 3 during zinc deficiency, activating cyclin-dependent kinase 2 (CDK2). Consequently, cells entered prematurely into S-phase and were subjected to apoptosis [92,161]. In addition to its intra-thymic activity, thymulin affects proliferation and activation of T cells in blood and tissue [162]. As zinc supplementation was able to restore thymulin activity in the thymus as well as in blood and tissue in humans and mice suffering from mild zinc deficiency, the reversibility of defects arising from zinc deficiency is probable and therapeutic zinc application should therefore be beneficial [163,164,165]. Interestingly, expression of MT-1 and calprotectin is increased in various cell types during differentiation. As both are important intracellular zinc binding proteins, this could increase the amount of zinc immediately available for signaling processes [80,166]. The latter might explain the manifold stronger reaction of mature cells to stimulation compared to immature cells, as the magnitude of the fast zinc signal is most probably much higher due to high MT levels. 

### 5.4. Zinc, Smad Signaling and the Development of Regulatory T Cells

The natural importance and therapeutic benefits of regulatory T cells (Tregs) in preventing auto-immune diseases and transplant rejection is becoming more and more accepted [167]. One important trigger for Treg development is TGF-β-induced Smad 2/3 signaling, which induces expression of the transcription factor forkhead-box-protein p3 (FoxP3), which is essential for lineage commitment. Zinc supplementation augmented TGF-β-induced Smad 2/3 signaling and thereby FoxP3 expression [168]. Proteasomal degradation of FoxP3 involves the histone deacetylase Sirtuin-1 (Sirt1), which is inhibited by zinc as was shown for other histone deacetylases as well [72,169]. Another important, zinc dependent molecule in Treg cell development is interferon regulatory factor (IRF)-1, as it is able to repress FoxP3 activity [170]. Zinc supplementation dampened IRF-1 activity, increasing tolerance. 

In addition to its role in Treg development, zinc also affected Treg function. Here, CK2-mediated activation of ZIP7 was of central importance [171]. The transporter ZIP7 is located in the endoplasmic reticulum and responsible for the release of zinc into the cytosol. Phosphorylation of ZIP7 by casein kinase II (CK2) activated it, causing a zinc flux, which was suggested to cause activation of signaling pathways, resulting in cell proliferation and migration [171]. Thus, zinc was able to induce and stabilize Treg cells in this study, underlining its therapeutic importance. 

Rice et al. investigated MT expression and efficiency of signal transduction in Type 1 regulatory T cells (Tr1), another recently described member of the family of Treg cells. Intracellular zinc and MT expression were increased during differentiation of naïve T cells into Tr1 cells after six days [172]. When IL-27 was also added, zinc increases and MT-1 expression was augmented. In addition, cells with higher MT levels more efficiently transduced a ROS signal into a zinc signal, as monitored via p38 MAPK signaling. MT overexpression was observed during chronic inflammatory diseases and cancer. The correlation of MT expression with efficiency of signal transductions might explain the high reactivity of cells during chronic zinc deficiency [172]. Whether this explains hyperinflammation, observed during sepsis-induced augmented serum hypozincemia, needs to be explored. Calprotectin is another zinc binding protein that is elevated during severe inflammatory disease and serum hypozincemia [63]. In contrast to MT, expression of calprotectin was recently shown to be induced by zinc deficiency, similarly to what has been described for pro-inflammatory cytokines [66,173].

### 5.5. PMA-Induced NET Formation in Granulocytes

A representative example illustrating nicely the importance of zinc in regulating cell signaling is the formation of NETs by granulocytes. ROS production in neutrophils via NADPH oxidase is an important prerequisite for NET formation. Both zinc deficiency as well as excess have been shown to inhibit superoxide anion production [174] and thereby the formation of NETs. When zinc was chelated in granulocytic cells, the PMA-induced increase in intracellular zinc was abolished, as well as NET formation [83], showing that the assumption of a tight zinc and redox metabolism interrelationship holds true for granulocytes and their functions. Whether other pathways are involved in NET induction remains to be investigated. 

### 5.6. TLR4-Induced Signaling: A Great Example of Fine-Tuning of Signaling Pathways by Zinc

**Figure 2:**
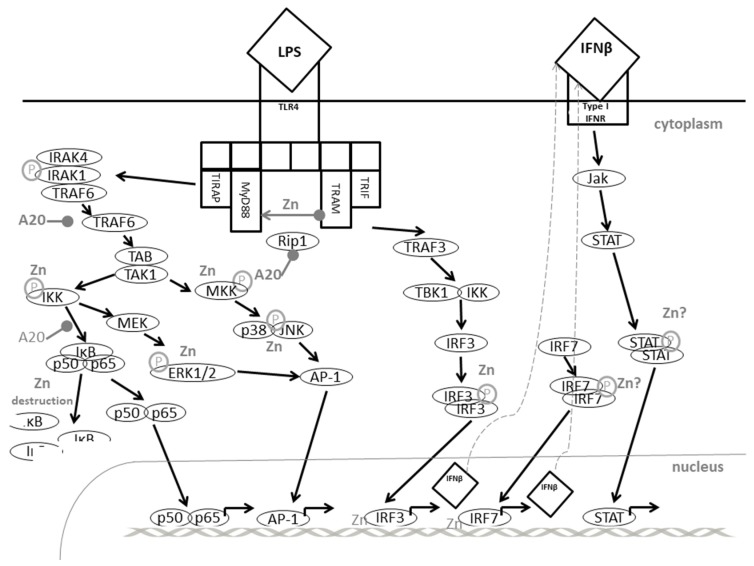
Zinc in Toll like receptor 4-induced signaling. For explanation, see text. Abbreviations: A20: zinc finger protein; AP-1: Activator protein 1; ERK: extracellular Signal-regulated Kinase; IFN: interferon; IRAK: Interleukin-1 receptor-associated kinase; IκB: Inhibitor of NFκB; IKK: IκB kinase; IRF: interferon related factor; JAK: JNK janus kinase; JNK: c-Jun *N*-terminal Kinase; LPS: Lipopolysaccharide; MAPK: mitogen activated protein kinases MEK: MAPK/Erk kinase; MKK: MAPK kinase; MKP: MAPK phosphatase; MyD88: Myeloid differentiation primary response gene; NFkB: nuclear factor (NF)κB; PI3K: phosphatidyl-inositol-3-phosphate; RIP: receptor interacting protein; STAT: Signal transducers and activators of transcription; TAB: TAK-binding protein; TAK: TGF β-activated kinase; TBK: Tank-binding kinase 1; TIRAP: toll-interleukin 1 receptor (TIR) domain containing adaptor protein; TLR: Toll like receptor; TRAF: TNF receptor-associated factor; TRAM: TRIF-related adaptor molecule; TRIF: Toll-interleukin-1 receptor (TIR) domain-containing adaptor-inducing interferon. Altered from [97,176,178].

Triggering of TLR4 at the surface of myeloid cells by LPS or other ligands activates several signaling pathways as illustrated in Figure 2. This is a great example to illustrate how zinc flux and homeostatic zinc signals are able to fine tune cell signaling. This example also shows how long-term changes in extracellular zinc resulted in different effects to those from acute alterations.

LPS binding to TLR4 caused a zinc flux, which subsequently inhibited dephosphorylation of MAPK and supported phosphorylation of IKKα/β so that NFκB translocated into the nucleus and activated expression of pro-inflammatory cytokines [94]. When zinc signals were prevented using chelators such as TPEN (*N,N,N′,N′*-Tetrakis(2-pyridylmethyl)ethylenediamine), activation of kinases ERK1/2, IKKβ, MKK3/6 and IκB was abrogated, as shown for murine macrophages. Zinc chelation blocked the destruction of IRAK1 causing its accumulation in the cytosol. In contrast, IRAK1 phosphorylation and ubiquitination were not affected shortly after LPS-triggering. This might be due to the inhibition of zinc-dependent MMPs, which are probably responsible for IRAK1 destruction during adequate zinc conditions. IRAK1 is responsible for the degradation of NFκB inhibitors by IKK. Therefore, NFκB did not translocate to the nucleus. Furthermore, ERK signaling was disturbed when zinc was chelated, which might be associated to the effect of zinc on PTP and PTK activities as described earlier [175,176]. 

The MAPK and NFκB pathways described above are all induced by TLR4 ligation via myeloid differentiation marker (MyD)88. In addition, MyD88-independent/Toll-interleukin-1 receptor (TIR) domain-containing adaptor-inducing interferon (TRIF)-dependent pathways involving TRIF-related adaptor molecule (TRAM), TRIF and interferon regulatory factor (IRF)-3, are also activated. In contrast to pro-inflammatory mediators, activated via MAPK and NFκB, IFNβ expression is triggered here, which functions in an autocrine fashion and activates the JAK-STAT pathway as well as a delayed translocation of NFκB to the nucleus after binding to the IFNR on the cell surface. This upregulates CD40, CD80, and CD86 expression enabling the cell to interact with T cells [97,177].

Furthermore, the inducible nitric oxide synthases (iNOS) is induced via the JAK-STAT pathway, resulting in NO production to support the anti-microbial reaction. During zinc deficiency, murine macrophages were shown to produce more NO after LPS stimulation than controls. Zinc deficiency also augmented LPS-induced expression of IFNγ, CD80 and CD86 in murine macrophages either bone marrow-derived, or from cell culture, while expression of IL-1β, IL-6 and IL-10 was decreased. All effects were normalized when zinc deficient cells were reconstituted with zinc prior to stimulation. Here, analyses were performed a few hours after LPS stimulation, representing homeostatic zinc signals. As incubation of cells with a zinc chelator after LPS-stimulation still had the same effects, one can conclude that effects on MyD88-independent pathways are not associated with the zinc flux, but rather by the homeostatic zinc signal [97].

The delayed favoring of the MyD88-independent pathways is paralleled by inhibition of parts of the MyD88-dependent pathways as well. Here, ubiquitinated TRAF6 activates IKK and MKK via the kinase TAK1. However, as mentioned earlier, ubiquitin residues are removed from TRAF6 by A20, highly expressed due to the homeostatic zinc signal, resulting in suppression of gene expression [148,179,180]. In addition to zinc, pro-inflammatory cytokines are known inducers for A20 in macrophages, possibly representing a feedback-mechanism to prevent hyperinflammatory reactions [181]. This fits well with the deregulated inflammatory response seen during chronic and severe serum hypozincemia, as observed during sepsis [63,65]. Finally, a role of zinc in TLR4-triggered signaling via zinc’s effect on cyclic nucleotides was suggested. In this connection, elevated cGMP could be responsible for increased Raf-1 phosphorylation, which inactivates this MAPK and also prevents NFκB activation and translocation. Signaling induced via other TLR might be regulated in a similar manner. Zinc-dependent regulation of TRIF/IRF/IFNβ signaling was observed as well when cells were stimulated with TLR3- or TLR7-specific agents [97]. 

Recently, a first approach to analyzing the zinc-dependents of the proteome was published. Here, macrophages were incubated with either zinc oxide nanoparticles or zinc acetate in equivalent doses. After at least one day, the proteome of the cells was analyzed using a 2D gel electrophoresis technique. The amount of several signaling molecules was altered by zinc incubation. Most interestingly, MyD88 protein levels were altered due to incubation with the nanoparticles or the zinc acetate [182]. Unfortunately, there is so far no information as to whether this is due to increased expression or decreased degradation of the protein and tests of MyD88 function were also not included, thus more analyses are needed. However, the results of this study suggest new targets that are probably affected by zinc signals and cellular zinc homeostasis.

Altogether, this is not only a great example of fine-tuning of signaling by zinc but also underlines the complexity of zinc’s role in intracellular signal transduction. 

### 5.7. Zinc Levels Regulate Fc Receptor-Induced Signaling

Instead of recognizing PAMPs, some immune cells are equipped with receptors for immunoglobulins (Ig) to detect immune complexes or opsonized pathogens. One example is the Fcε receptor (FcεR) on mast cells, whereby ligand binding causes degranulation and thereby the release of anti-microbial agents. Those granules are also known to contain high amounts of zinc, released during degranulation, probably causing zinc intoxication of pathogens in close proximity to the immune cell. The role of zinc in mast cells and their signaling has been poorly investigated, but some data indicate that triggering the FcεR causes a zinc wave via release of zinc from the perinuclear area, such as from the endoplasmic reticulum (ER) [96,183]. Thereby, PKC was transported to the plasma membrane, ERK1/2 and JNK1/2 were phosphorylated and NFκB was activated and translocated to the nucleus, inducing expression of pro-inflammatory cytokines [96,184]. When the zinc wave was prevented by incubation of the cells with TPEN, signaling and gene induction were blocked. In line with this, signaling was prolonged when zinc was added to the cells. In addition, zinc seems to be important for fusion of the granules with the plasma membrane, thus enabling degranulation [184]. Zinc chelation inhibited degranulation. Within these studies, two pools of zinc were identified by using different zinc probes: the larger pool of granular zinc and the smaller, variable pool of cytoplasmic zinc. This emphasizes that individual probes can vary in their intracellular location, also depending on the cell type and its activation state [71,185,186]. 

### 5.8. Killing Activity of NK Cells Varies with Zinc Availability

NK cells are able to destroy transformed or infected cells, largely due to recognition of changes in major histocompatibility complex MHC-I composition that are caused by the degeneration or the pathogen. In contrast, regular cells are protected from killing by NK cells as long as they carry surface MHC-I, which is bound by the killer cell inhibitory receptor (KIR) on NK cells [187]. To form the so-called NK cell synapse, KIR multimerization is essential, and was shown to be zinc-dependent [87,89]. Unfortunately, there are no data on complex formation during zinc deficiency, but a disturbance in zinc homeostasis would explain why killing is reduced if zinc supply is limited and increased if additional zinc is supplied [88]. Some recent data suggest a zinc signal after NK cell activation with IL-1β [188]. IL-1R signaling is known to be affected by zinc as will be described later. Because NK cells carry a variety of surface receptors containing tyrosine phosphorylation sites, including immunoreceptor tyrosine-based inhibitory motif (ITIM) and immunoreceptor tyrosine-based activation motif (ITAM), a role for zinc in signal transduction is likely, and this possibility should be tested.

### 5.9. The Role of Zinc in IL-2-Induced Signaling in T Cells

When IL-2 binds to its receptor, signal transduction via Jak1 and Jak3 is initiated and the adaptor Shc is recruited. Oligomerization of the receptor is essential for its function, thus the IL-2R subunits are found in lipid rafts. Subsequent to IL-2 stimulation, three different pathways can be activated, centering around PI3K/Akt, STAT5 and MEK1/2-ERK1/2. IL-2 is known to induce proliferation and development of effector functions in T cells, inducing anti-apoptotic and cell-cycle related genes as well as certain cytokines and lineage decisive factors [189]. 

Similar to the effects of other growth-promoting factors, binding of IL-2 to its receptor induces a zinc flux. Here, zinc is released from lysosomes and zincosomes, which was linked to ERK1/2 and Akt activation, but did not affect STAT5. In this context, Phosphatase and tensin homologue deleted on chromosome 10 (PTEN) is of importance, as it degrades PI3K-produced PI(3, 4, 5)P3, which subsequently activates Akt. The zinc IC_50_ of PTEN was found to be 0.59 nM, which would allow its blockade by zinc flux, explaining activation of Akt [71,135]. 

### 5.10. Zinc in T Cell Receptor Signaling

**Figure 3:**
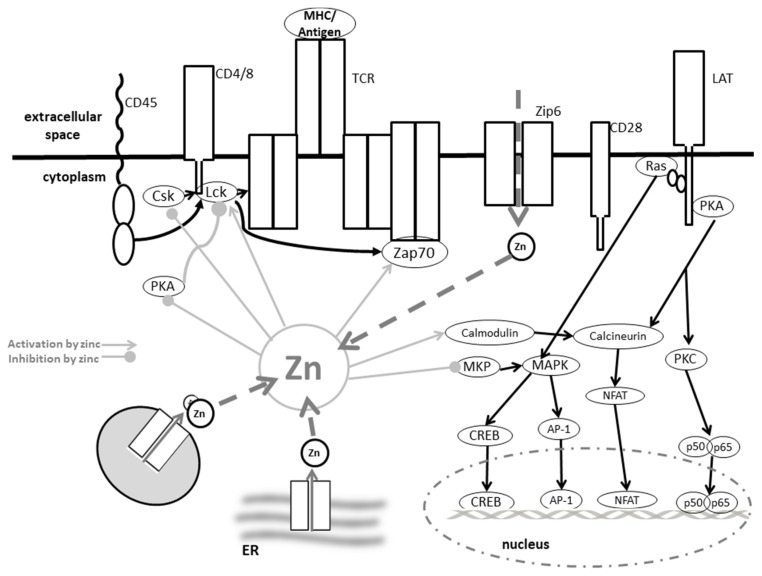
Zinc in TCR-induced signaling. For explanation, see text. Abbreviations: AP-1: Activator protein 1; CREB: cAMP response element-binding protein; Csk: C-terminal Src kinase; ER: endoplasmic reticulum; Lck: lymphocyte-specific protein tyrosine kinase; LAT: linker for activation of T cells; MAPK: mitogen activated protein kinases; MHC: major histocompatibility complex; MKP: MAP kinase phosphatase; NFAT: Nuclear factor of activated T cells; p50/p56: nuclear factor NF-kappa-B subunit p50/p65; PKA: protein kinase A; PKC: protein kinase C; Ras: Rat sarcoma; TCR: T cell receptor; ZAP: z-chain-associated protein kinase; Zip: Zrt-like, Irt-like proteins. Modified after [109,170].

The major function of the T cell receptor (TCR) is the specific recognition of a certain antigen, presented by an MHC molecule. As the TCR lacks intrinsic kinase activity, the CD3-complex, responsible for signal transduction, needs to be recruited and non-covalently bound to form the functional receptor. PTKs, involved in the subsequent highly organized signaling cascade include members from Src, Syk, Csk (C-terminal Src kinase) and Tec families. In addition, certain adapter proteins and effector enzymes are involved, as illustrated in Figure 3. Activation of the signaling molecules via phosphorylation by PTKs can be rapidly reversed, as PTPs have a higher capacity than the PTKs. Genes expressed after TCR triggering are dependent upon the stimulus and co-stimuli characteristic of a certain T cell subsets, causing proliferation and differentiation [109].

The importance of zinc as an ionic signaling molecule after TCR triggering has been investigated in various studies. A zinc flux was measured within less than one minute after ligand binding to the TCR. As silencing of ZIP6, located in the plasma membrane, prevented this fast zinc signal, the origin of the zinc seems to be extracellular [190]. When extracellular zinc was depleted from the media, no fast zinc signal occurred after TCR triggering, supporting this hypothesis. Subsequent to its fast increase, zinc is compartmentalized into a subsynaptic region, which is quite different from the calcium wave, which originates from intracellular stores and spreads rapidly throughout the cell [191]. However, in addition to extracellular zinc, the fast zinc signal might also be caused by ZIP8-mediated release of zinc from lysosomes, recruited to the TCR complex after stimulation [101]. In response to certain environmental conditions, basal expression of MTs was increased. This rendered more releasable zinc available for subsequent stimulation. Consequently, lower levels of MTs observed during prolonged zinc deficiency could explain the lower response of T cells to antigen, as less zinc for a zinc flux would be immediately available. 

Targets of this zinc flux have been found within several stages of signal transduction after receptor triggering. As has been described above, the TCR alone is not able to activate a signaling cascade. Therefore, CD4 brings the Src kinase Lck (lymphocyte-specific protein tyrosine kinase) into proximity with the TCR, thus enabling tyrosine phosphorylation of signaling molecules, including ZAP70 and CD3ζ. Zinc facilitated the binding of Lck to CD4, as it did to CD8, wherefore it is of central importance for complex assembly and the initiation of the signaling process [192].

Not only recruitment, but also activity of Lck, were affected by zinc, as it enabled Lck homodimerization by stabilizing the SH3 domain [193]. Lck activity can additionally be inhibited by phosphorylation of tyrosine Y505, regulated by the PTP CD45 and the kinase Csk [194], or activated by phosphorylation of Y394, which is dephosphorylated by PTPN22. A direct effect of zinc on the activity of phosphatases and kinases was described [195]. Zinc supplementation of T cell lysates did indeed result in dose-dependent decrease of total phosphatase activity [71]. In support of this, Csk is inhibited by zinc [196], as probably is PTPN22. Moreover, recruitment of PTPN22 is regulated by Csk [102]. A direct effect of zinc on CD45 activity has been found in vitro, when zinc was supplemented in high doses, and this remains to be investigated in more detail [103]. As Lck is responsible for the phosphorylation of ZAP70, which then further activates signaling molecules such as MAPK, abrogation of the initial zinc flux can have far reaching consequences [71,197,198]. 

Further downstream, zinc might also affect transcription factor binding. One important target is NFAT, responsible for IL-2 expression, but retained in the cytoplasm of resting T cells due to constitutive phosphorylation. After TCR triggering, it is dephosphorylated by CN, serine/threonine phosphatase [199]. As physiological concentrations of zinc between 10 nM and 10 µM were able to inhibit CN activity, NFAT translocation might also be affected by the zinc flux. However, no direct evidence is available and, as the zinc signal seems to stay local, i.e., close to the receptor, this needs to be further investigated. In addition, regulation of CN activity is rather complex. CN itself is constitutively inactivated by phosphorylation. Here, phosphatidyl-inositol-3-kinase (PI3K) is responsible. PI3K activity is also zinc sensitive. Zinc supported enzymatic activity via effects on Akt and PTEN explored in detail for IL-2-induced pathways [135]. If effects are similar for TCR-induced signaling, needs to be explored. 

In this regard, consequences of homeostatic zinc signals will be described, as they also affect CN activity. TCR triggering causes upregulation of ZIP8 expression. Therefore, zinc is released from the lysosomes causing an increase in intracellular zinc, when the zinc flux has probably already vanished. This homeostatic zinc signal has been connected to a blockade of CN activity which results is transcription of IFNγ [101]. Another consequence of the homeostatic zinc signal is augmented PKC activity due to facilitated binding of phorbol esters and higher affinity to cytoskeleton and plasma membrane [119]. As a final note on the importance of zinc for TCR-triggered T cell response, the activation threshold for stimulation was lowered when additional zinc was supplemented to the cells. Thus, even suboptimal antigen conditions caused T cell proliferation [102], which might be interesting for optimizing vaccination. As a note, calcium influx was unaltered, when zinc conditions were changed. 

When T cells are activated, MT expression increases, which was associated to decreased intracellular zinc and ROS levels and a sustained proliferation of the T cells [101]. Thus, also for TCR-induced signaling, effects of zinc further downstream are likely. As the zinc flux increased MT expression in the T cells, not only immediate signaling pathways were affected, but T cell proliferation and survival were supported in the long term [200]. In addition, TCR ligation was also followed by ROS generation, known to provide zinc from protein for signaling regulation [201]. Triggering of TCR led not only to increased activity, but also to an increased expression of ZIP6 [202]. The influx of extracellular zinc inhibited the recruitment of SHP-1. As SHP-1 is a negative regulator of TCR-signaling, this might function as a feedback mechanism to end TCR-mediated signaling [102]. The lower recruitment of SHP-1 might either be due to changes in its conformation or the serine phosphorylation of Lck, which prevents SHP-1 binding.

### 5.11. The Role of Zinc in IL-1 Receptor Signaling in T Cells

IL-2 functions as a majorly proliferative agent for T cells, whereas other cytokines, such as IL-1 also induce the expression of inflammation-related genes such as IFN-γ, in addition to supporting proliferation. After IL-1R triggering, IL-1 receptor-associated kinase (IRAK) is activated, causing NFκB translocation to the nucleus, culminating in gene activation. Here, zinc supplementation of T cells was shown to decrease IRAK activity, similarly to its decreasing TLR4-induced signaling pathways, resulting in repression of the memory Th17 response [97,203]. It has been reported that IL-1 binding causes a zinc signal [204]. MyD88 is another central signaling molecule of IL-1-induced signaling, and a role for zinc in this pathway has been described [97]. Effects have not been explored for the IL-1R, yet.

The last paragraphs underline the complexity of zinc’s effect on T cell development, lineage decision and activity. As effects were described for each pathway individually, this is an oversimplification of the in vivo situation. In addition, effects of for example zinc supplementation and zinc deficiency on the outcome of the investigated T cells will also depend on other environmental factors and the cellular environment, rendering prediction of an overall effect impossible. 

### 5.12. The Role of Zinc Signaling in B Cells

When B cells are mentioned, antigen recognition and antibody production come to mind. Antigen recognition, and thereby choice and activation of a certain B cell clone, is mediated via the B cell receptor (BCR). BCR activates almost the identical intracellular pathways to TCR. These pathways focus, amongst others, on the same kinases (PKC, MAPK) and transcription factors (NFAT, NFκB) [108]. Surprisingly, the correlation between zinc status and the proliferation and overall functions of B cells was much weaker than for T cells, as was shown by investigations in mice [205,206]. A considerable difference was found between the effects of acute and chronic zinc deficiency: While acute zinc deficiency caused a reduction in overall B cell numbers, this was far less pronounced during chronic zinc deficiency [207]. Recently, the regulation of ZIP10 expression by STAT3 and STAT5 was revealed and associated with suppression of apoptosis in human B cell lymphoma, suggesting some effects of homeostatic zinc signals on the development and survival of B cells.

Generally, binding of antigen alone is not sufficient to activate the complete maturation of a B cell into an antibody-producing plasma cell. Recent studies indicate that ligation of the B cell-activating factor receptor (BAFFR), TLRs and CD40, synergize with the BCR to define the repertoire of the mature B cell [208]. Additionally, co-stimulation by T cells is required. As T cell numbers and activation are strongly compromised during zinc deficiency, zinc was first suggested to play an indirect role. This was in line with the observation that especially T cell-dependent antibody production was affected [209]. However, antibody production per cell was largely constant under zinc deficient conditions [210]. To circumscribe effects due to alteration of T cell numbers and functions, Hojyo et al., in an elegant study, blocked the zinc signal in B cells, while not affecting the T cell compartment [211]. The group identified ZIP10 as being responsible for zinc signals in antigen-presenting cells, including B cells. To specifically knock out ZIP10 in antigen-presenting cells (APC), the ZIP10 gene was conditionally ablated by being put under the control of the invariant chain promotor. The invariant chain gene is constitutively activated in APC, but not in other cells including T cells. Interestingly, BCR-induced signaling was severely disturbed in cells from ZIP10 knockout mice and production of specific immunoglobulin (Ig)M and IgG was strongly decreased, which suggest that zinc indeed plays an important role in BCR-induced signaling. Interaction with T cells usually induces the germinal center (GC) reaction necessary for Ig class switch in B cells. Formation of GC B cells was also reduced in cells from ZIP10 knockout mice. BCR-crosslinking-induced proliferation was diminished, although hyperactivation of ERK, AKT and NFκB was observed and activity of SYK and LYN was increased. Only CD45 activity was less than half in cells from ZIP10 knockout mice compared to ZIP10 expressing controls [211]. In general, those observations are in line with what has been observed in zinc-deficient animals, suggesting that not only indirect effects via the disturbed T cell compartment are responsible for the defect in B cell response. Of note is that TLR-induced signaling was not affected by ZIP10 knockout. As the origin of zinc for TLR-triggered zinc signals is largely protein and organelle-based, this is also in line with previous findings [94,98,212]. 

In addition to BCR-induced signaling, pathways in B cells affected by zinc included those centered on STAT3 and STAT6, activated for example by IL-6 or IL4, respectively. Here, in vitro studies revealed that IL-4-induced STAT6 and IL-6-induced STAT3 phosphorylation were decreased during zinc deficiency [145]. As the IL-4-induced pathway is essential for the immunoglobulin class switch to IgE, this might explain why susceptibility towards especially parasite infections is increased under low zinc conditions [145]. IL-6 is essential for the induction of terminal B cell development into plasma cells [213], providing an additional reason for the decrease in antibodies during zinc deficiency. On the other hand, IL-6 is known to induce the production of autoantibodies if plasma levels are highly elevated. Thus, the association of IL-6, STAT3 phosphorylation, and (auto-) antibody production needs to be investigated more closely.

Furthermore, zinc deficiency was shown to increase apoptosis in B cells during early developmental stages, similar to what was described for T cells [214]. Fewer naïve B cells were found during zinc deficiency, while numbers for precursor B cells and cycling pro-B cells were not largely affected. While the response to known antigens was still efficient, reaction to neoantigens by B cells declined during zinc deficiency. This might be one reason for the decreased efficiency of vaccination in the elderly, who are known to be at least marginally zinc deficient in most cases [215,216]. Along with this, zinc uptake into cells through the zinc transporter ZIP10 was essential for early B cell development, increasing cell survival [217]. Others found that the effect of zinc on apoptosis via caspase activity depends on the dosage: treatment of Burkitt Lymphoma cells with up to 50 µM of zinc inhibited caspase 3 activation and apoptosis, whereas higher concentrations up to 100 µM rather induced programmed cell death via caspase 3 [218].

Homeostatic zinc signals affected various stages in apoptotic signaling cascades, including production and activity of members of the Bcl/Bax family [214]. In addition, DNA fragmentation was decreased when sufficient zinc was available, as the endonuclease responsible for this process is calcium dependent but inhibited by zinc. Furthermore, caspases 3, 6 and 8 were inhibited by zinc in a dose-dependent manner. IC_50_ for caspase 3 was for example 10 nM zinc, which is within the physiological intracellular range [137,219]. These three caspases are known mediators within the apoptotic pathway [220].

## 6. Zinc and Transcription Factors

In the early 1980s, zinc binding sites were found in *Xenopus laevis* transcription factor IIIA (TFIIIA) 24. This discovery offered new approaches to discover structural zinc binding sites in other species similar to those uncovered in *Xenopus laevis*. Thus, for the first time the typical zinc-finger structure was defined in TFIIIA and consisted of nine repetitive sequences containing cysteine (C) and histidine (H) residues. Since then, the discovery of zinc-binding sites dramatically increased within a short period, based on homology searches in sequence databases for the characteristic ligands in TFIIIA. Today, nearly 3000 proteins with zinc-binding motifs have been identified by bioinformatic approaches, making up ~10% of the proteins encoded within the human genome potentially regulated by zinc [43]. 

Regarding the human immune system, the innate and adaptive immune system are both regulated by zinc-finger-bearing transcription factors. Hence, a direct as well as an indirect role of zinc in altering intracellular signaling can be anticipated. During the development of innate immune cells, essential transcription factors such as GATA-4/-5/-6 and KLF-4/-5 are potentially zinc-regulated targets since they contain a zinc-finger domain [221,222]. Moreover, the promyelocytic leukemia zinc-finger is a critical transcription factor for iNKT cell development. In myelopoiesis, expression of the transcription factor PU.1 is decisive for lineage commitment [223]. With respect to the adaptive immune system, a multitude of zinc-finger-containing transcription factors have been identified. In T cell development, various transcription factors are important for the adequate differentiation into CD4^+^ T cells, such as the zinc-finger transcription factors GATA-3 and Zbtb7b (Thpok, cKrox) [224,225]. Moreover, GATA-3 is essential for Th1 and Th2 subpopulation differentiation [226,227]. Generally, the zinc-finger-containing KLF family has an important function in immune cell differentiation, for example KLF-2/-3 in B cells, KLF-2/-13 in NKT cells, and KLF-2/-4/-10 in T cells [228]. Consequently, it is not surprising that Treg differentiation is driven, amongst other things, by zinc-dependent KLF-10 modulation [170]. 

Concerning the overall regulation of zinc homeostasis, numerous genes exhibiting metal response elements in their promotors are involved. These can be induced by binding of the metal-response element-binding transcription factor (MTF)-1 that itself is regulated by the surrounding available free zinc concentration. Thus, MTF-1 acts as an intracellular zinc sensor, leading to the suggestion that feedback mechanisms may exist [229]. The field of zinc-regulated transcription factors is still under investigation and offers great opportunities for further research to better understand modulation of the immune function due to zinc supplementation or zinc deficiency, respectively. Regulation of transcription factors by zinc homeostasis is a good example of how a single element such as zinc can balance the immune response and therefore acts as a “gatekeeper”. 

## 7. Zinc, Epigenetics, and Immunity

Over 75 years ago, Waddington chose the term “epigenetics” to describe changes to DNA other than in its sequence that were able to alter gene expression [230]. Those non-DNA-encoded changes include mainly DNA methylation (methylation of cytosine bases) and modification of histone tails by adding chemical groups such as one or more acetyl, methyl, or sumoyl residues. Alterations cause nucleosome re-positioning and expose or block binding sites for transcription factors or suppressors, thereby promoting or inhibiting the expression of adjacent genes [231]. Non-coding RNAs and transcription factor regulatory networks also count as epigenetic mechanisms. During recent years, the field of nutritional epigenetics emerged, covering the effects, amongst others, of nutritional elements such as zinc, but also barium, calcium, chromium, manganese, magnesium, iron, selenium, sodium, molybdenum, phosphorus, potassium and sulfur on the epigenome [232,233]. 

The finding that the establishment of the epigenome starts as early as during oocyte development, clearly underlines the importance of a well-balanced nutritional and mineral supply during pregnancy and breast-feeding. Zinc supply influences health and development from early in life, into old age [234]. In addition, lifestyle-dependent changes in the epigenome can occur throughout life. Dysregulations within the epigenome have been observed to accumulate in the elderly [235]. Epigenetic changes can even be carried over to the next generation, as has been shown by experiments with rats [236]. Various environmental factors, including: (1) individual characteristics such as age, gender and genetic predisposition; (2) lifestyle-related factors including the amount of exercise, smoking, and stress; and (3) nutrient-related factors such as dose, route, duration of the exposure as well as the presence and variety of elements, affect the epigenome and are summed up as “interactome”. However, the interactome is difficult to estimate, as already drinking water and soil might contain different amounts of each trace element depending on the region [233]. Dysplasia and disease originating from all developmental stages, including diabetes, multiple sclerosis and other mainly auto-immune and neurological diseases have so far been connected to epigenetic abnormalities, caused by nutritional dysbalance, as will be exemplified later regarding zinc [233,237,238].

As indicated earlier, zinc is an important co-factor for enzymes from all classes of enzymes. This includes epigenetically-active enzymes such as class I, II, and IV histone deacetylases (HDACs) [239] and acetylases (HATs), DNA methyltransferases (DNMTs), histone methylases and methyl-binding proteins. Zinc deficiency has been associated with global DNA hypermethylation [240]. One explanation is the effect of zinc on activity of methionine synthase. This enzyme is responsible for the regeneration of methionine from homocysteine, providing substrates for DNA and histone methylation [241].

Disturbance of bone development during zinc deficiency has been shown to be epigenetically determined. Indirect effects of zinc on the epigenome include the multi-zinc-finger enzyme Trps1, named after the “Tricho-Rhino-Phalangeal syndrome”, where it is deregulated. During mitosis Trps1 regulates histone deacetylation in a zinc-dependent manner. In addition, it increases the activity of HDAC1 and HDAC2, leading to histone H3 hyperacetylation if *Trps1* is non-functional. As a consequence, prometaphase cells accumulate due to impaired HP1 binding and chromatin condensation in Trps1-deficient chondrocytes [242]. Trps1 might be involved in regulation of mitosis in T cells, which needs to be explored in more detail and would offer another explanation for the dependents of hematopoiesis on zinc homeostasis.

As is true for most trace elements, zinc deficiency during pregnancy was associated with abnormalities in development of the embryo and poor health of the newborns, amongst others due to disturbances in immune cell development [243,244]. Here, various studies revealed that effect on development depends on the stage at which zinc deficiency was present. Regular development of the oocytes was paralleled by increasing methylation of their chromatin. Moreover, zinc was required to complete meiosis, as was shown for oocyte development in vitro and in vivo [236]. If the oocyte was zinc deficient after fertilization, meiosis failed due to disorganized chromatin, which should also be investigated in immune cell development. Various pathologies, such as chronic inflammatory diseases that appear later in life, have been associated with zinc deficiency during pregnancy. In addition to hypertension, autism and impaired learning, persistent immunodeficiency was observed for three subsequent generations in rats [245,246,247,248]. Interestingly, the regulation of genes, involved in zinc homeostasis was also affected by epigenetic mechanisms. Zinc-dependent epigenetic changes in the MT-2 gene in utero caused significantly higher MT-2 expression in the liver later in life as observed in mice [249]. Similarly, not only the function, but also the expression of zinc-finger proteins, including ZNF804A and ZNF326, was affected by the zinc status via epigenetic mechanisms [250,251]. As found during investigations in autistic patients, zinc deficiency during pregnancy caused epigenetic abnormalities in genes encoding MTF1, ZnT5, COMMD1 (COMM domain-containing protein 1), ERK1, TrkB (tyrosine-related kinase B), and ProSAP/Shank (proline-rich synapse-associated protein/SH3 and multiple ankyrin repeat domains) [252,253,254,255]. As those are important players regulating immune cell development and function, investigations on similar effects in immune cells are warranted. 

Chronic inflammation, but increased susceptibility to infections are a typical symptom of aging. Amongst others, the accumulation of unusual epigenetic changes during aging was suggested to be responsible for altered activation of immune cells, causing those symptoms. Basal expression of inflammatory mediators such as IL-1β, IL-6 and TNFα was increased in vitro, largely due to changes in chromatin structure of the corresponding genes [66,185]. As the expression of zinc transporters was also altered via epigenetic modifications, this creates a self-reinforcing positive feed-back loop [256,257]. In particular, ZnT5, ZnT1, and ZIP6 expression was affected, causing a decline of zinc in the elderly [241]. The reversibility of epigenetic changes if zinc is supplemented needs to be explored.

## 8. Zinc Deficiency in the Context of Disease

**Figure 4:**
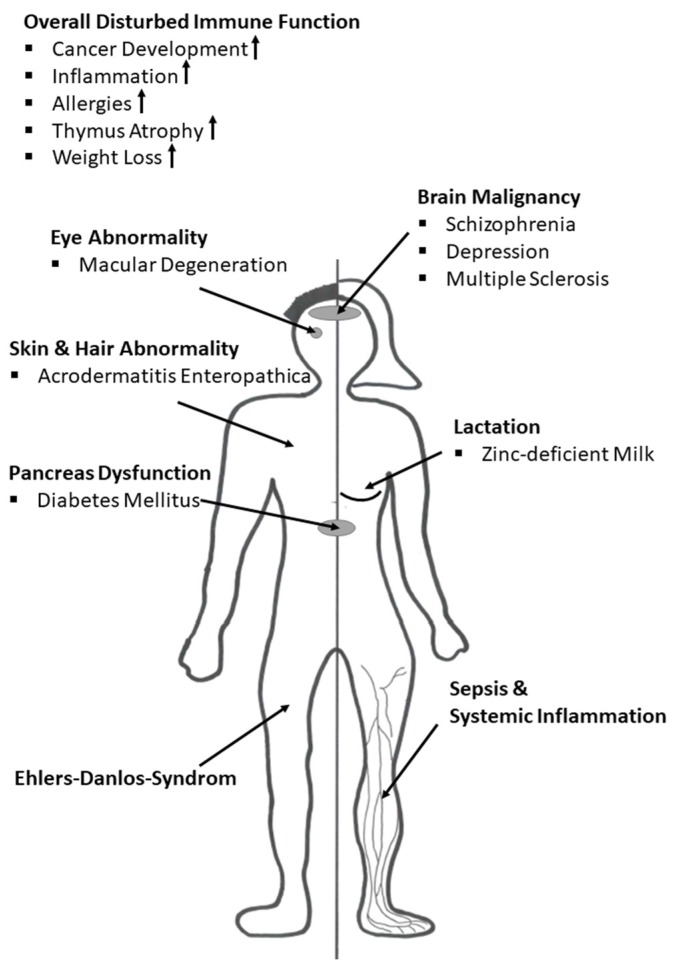
Influence of zinc and zinc deficiency on various organ systems as well as on the immune system. Zinc deficiency is causally associated with multiple immunological dysfunctions that lead to the manifestation of various diseases indicated in this figure. For a detailed explanation see the text. Modified after [5]. ↑ = upregulted/enhanced.

Zinc status is strictly regulated by the zinc transporting proteins ZIP and ZnT. Malfunction of either ZIP or ZnT causes tremendous immunological impairment. Altered zinc homeostasis in the context of clinical manifestation of disease is summarized in Figure 4. 

### 8.1. Zinc and Acrodermatitis Enteropathica

Disruption of the ZIP4 gene was reported to impair early embryonic development in mice. This was due to malfunction of appropriate zinc transport into the developing embryo via the visceral yolk sac and to insufficient zinc uptake in the intestine, resulting in severe zinc deficiency. Similar effects of a non-functioning ZIP4 transporter and subsequent zinc deficiency were observed in humans suffering from Acrodermatitis Enteropathica (AE), as discussed above [47,258]. Patients suffering from AE showed several immunological alterations, such as thymus atrophy as well as decreased lymphocyte counts and lymphocyte function. This was not surprising, since the function of virtually all immune cells strictly depends on zinc homeostasis. Especially T cell function and maturation are highly zinc dependent, as described later in detail. Hence, AE patients have an increased susceptibility to infection, especially intercurrent bacterial, fungal, and other opportunistic infections are prevalent. Moreover, chronic diarrhea and lactose intolerance are also known, and without therapy AE is in general lethal. However, dietary zinc supplementation reversed the symptoms in both mice and humans [17,47,259]. T cell restricted malfunctions as well as abnormal chemotaxis were completely corrected by zinc supplementation.

### 8.2. Zinc in Cancer Development

The zinc transporter ZIP4 was shown to have an important role in cancer development. Microarray analysis identified steady state levels of ZIP4 mRNA to be highly upregulated in pancreatic cancer [260]. Biopsy analysis uncovered elevated ZIP4 mRNA expression in tumor samples as well as overaccumulation of ZIP4 protein [261]. Cell culture analysis determined that ZIP4 overexpression in human pancreatic cells increased zinc accumulation and cell proliferation respectively showing a connection between zinc status and cancer progression. Comparable results were obtained in experiments with mice. ZIP4 overexpression increased tumor weight as well as pathological fluid accumulation (ascites incidence). Additionally, increased zinc accumulation was apparent, as was a higher rate of cellular proliferation [262]. Knockdown of ZIP4 gene expression by siRNA inhibited tumor growth significantly and increased the survival rate of the mice [263]. Interestingly, increased ZIP4 expression was additionally found in hepatocellular carcinomas [264], emphasizing a potential role for ZIP4 in tumor development in general. At the molecular level, it was supposed that zinc transport via ZIP4 influenced cAMP response element-binding protein (CREB) activity, controlling numerous factors important in cellular proliferation [263,265,266]. Moreover, ZIP4 probably suppresses apoptosis and increases in vitro tumor cell migration [264].

In regard to breast cancer, the zinc transporters ZIP6, ZIP7 and ZIP10 were shown to be mainly involved in growth and invasive behavior. ZIP6, a member of the LIV-1 subfamily of ZIP proteins, was found to be estrogen-responsive and can therefore be used as biomarker for estrogen-receptor-positive cancers [267,268]. Additionally, luminal A-type breast cancer can be identified by ZIP6 expression [137]. Besides ZIP6, ZIP7 were highly abundant in breast cancer and were required for increasing the intracellular free zinc level [269,270]. ZIP10 expression was associated with breast cancer metastasis in lymph nodes and was strongly linked to invasive behavior [271]. Thus, ZIP6, ZIP7, and ZIP10 expression contributed to cancer cell survival and cancer progression [272,273]. At the molecular level, E-cadherin expression was modulated by ZIP6 as well as epidermal growth factor receptor (EGFR), Src, and insulin-like growth factor 1 receptor (IGF-1R) signaling molecules by ZIP7. Hence, cancer growth and invasive behavior is supported [274]. More recently, studies indicated a direct connection of phosphorylation of ZIP7 to be important for zinc efflux from intracellular zinc stores into the cytosol, leading to an elevated cytoplasmic free zinc level [98,275]. Consequently, signaling pathways such as mitogen-activated protein kinase (MAPK) signaling and tyrosine kinase activity were altered, leading to modulation of kinases and promoting aggressive breast cancer behavior. Amongst others, GFR, Src and IGF-1R signaling molecules were affected by zinc [269,276,277]. Cancer cells were highly apoptosis resistant due to an elevated intracellular zinc level, since caspase activity was found to be zinc-regulated [214]. Thus, killing of cancer cells by NK cells and cytotoxic T lymphocytes (CTL), respectively, was compromised. Moreover, a zinc-rich milieu induced Treg, impairing the anti-tumor response by inducing tolerance. Treg secreted high amounts of anti-inflammatory cytokines such as IL-10 and transforming growth factor (TGF)-β1 [278], constraining immunological anti-tumor defense. 

### 8.3. Zinc and Schizophrenia

It was reported recently that nonsynonymous variants in both ZIP8 [279] and ZIP12 expression [280] were associated with schizophrenia. The brains of patients suffering from schizophrenia showed lower zinc concentration than healthy controls [281]. In general, ZIP12 has a conserved function in vertebrate nervous systems, being responsible for proper development. A correlation between a homozygous missense mutation in the *ZIP12* gene and the frequency of schizophrenia was detected and claimed as a possible explanation [37,282]. Altered expression of ZIP12 was linked to congenital malformations caused by zinc deficiency [280]. Since T cell maturation, proliferation, differentiation, and function are highly zinc-dependent, and zinc deficiency impairs Th cell function [45,71,72,283,284], schizophrenia might be triggered due to malfunction of Th differentiation and function.

### 8.4. Zinc and Depression

Depression is a psychiatric disorder associated with high morbidity and mortality, being the cause of about 50–70% of all suicides [285]. The World Health Organization has predicted that depression will be the second leading contributor to the global burden of disease by 2020 [286]. Common symptoms for depression are sadness, exhaustion, and lack of interest in the daily activities of living [287]. 

In patients suffering from depression, increased pro-inflammatory cytokine concentrations, such as IL-1β, IL-6, and IFNγ, were found [288]. Furthermore, serum hypozincemia was observed, which was normalized by effective anti-depressant treatment. Interestingly, high amounts of zinc are present in the brain, particularly in the hippocampus and cerebral cortex [26,289]. It was shown that zinc deficiency negatively influenced brain zinc homeostasis and resulted in impaired mental function, altered behavior, learning, and susceptibility to epileptic convulsions [287,290]. Interestingly, zinc administration exhibited antidepressant-like effects when tested for antidepressant activity. Zinc administration produced antidepressant-like effects in the “forced swim test”, in both mice and rats, and in the tail suspension test [287]. During anti-depressant therapy in humans, Treg cells were induced while IL-1β level decreased concurrently [291]. Zinc administration induced Treg cells [72,170], leading to a dampened pro-inflammatory immune reaction. Thus, zinc deficiency can favor the development of depression, whereas zinc administration can exhibit antidepressant-like activity.

### 8.5. Zinc and Multiple Sclerosis

Worldwide there are about two million patients diagnosed with Multiple Sclerosis (MS), making it the most common chronic-inflammatory disease of the central nervous system (CNS) [22]. MS is characterized by: (1) inflammation of the CNS; (2) destruction of the myelin sheath, which surrounds the neuronal axon and forms an electrically insulating layer that is essential for proper signal transmission and thus for the function of the nervous system; and (3) paralysis, mediated by myelin-specific CD4^+^ Th cells.

One possible hypothesis for MS progression is believed to be due to T cell infiltration into the CNS boosting inflammation. Interestingly, T cell function is strictly regulated by zinc concentration, and zinc deficiency caused malfunctions and altered T cell differentiation, leading to an imbalanced immune response. Indeed, failures in zinc homeostasis were associated with the development of MS. In this regard, dietary zinc deficiency, as well as increased zinc uptake, were shown to be link with increased risk of MS and the development of pathogenic pro-inflammatory T cells in experimental autoimmune encephalomyelitis (EAE) [20,292,293]. Patients suffering from MS displayed lower plasma zinc levels and lower zinc concentrations in chronic MS lesions [21,22,294]. Zinc administration in EAE diminished EAE symptoms by inducing Treg cells and diminishing Th17 cells. Thus, the pro-tolerogenic immunoreaction was favored while the pro-inflammatory was dampened. In line with that, zinc signals are well known to be of central importance in keeping unwanted T cell-mediated immunoreactions in check, by suppressing apoptosis, immune cell proliferation, and pro-inflammatory cytokine production as well as IL-2, IL-10 and IL-17 production [214,295,296,297,298]. Therefore, adding zinc supplementation to current therapeutic standard treatments can be considered as potentially effective for patients suffering from MS. 

### 8.6. Zinc and Ehlers-Danlos Syndrome

Ehlers-Danlos syndrome (EDS) is a human disorder characterized by joint hypermobility, hyperelasticity of the skin, progressive kyphoscoliosis, and severe hypotonia of skeletal muscles [299]. The zinc transporter ZIP13 plays crucial roles in bone, tooth, and connective tissue development. Disturbed zinc homeostasis due to ZIP13 malfunction appeared to contribute to EDS pathogenesis, whereby the mutant ZIP13 protein was quickly degraded [300,301]. 

In addition to the identification of the EDS/ZIP13 relationship in humans, ZIP13 knockout mice were investigated. Those showed an abnormal systemic growth, bone growth, abnormal cartilage development, reduced dentin and alveolar bone, abnormal craniofacial and decreased dermal and corneal stromal collagen. At the molecular level, the absence of ZIP13 provoked a dysregulation of bone morphogenetic protein (BMP)-mediated gene expression [302]. Since BMP signaling covers a high variety of target genes, bone, tooth, and craniofacial development was impaired. Both BMP and transforming growth factor (TGF)-β signal via the TGF-β receptor and activate Smad signaling molecules containing a zinc-binding motif in the DNA binding domain, thus making Smad signaling a potential target of zinc signaling [303]. Therefore, ZIP13 might influence zinc availability for that motif.

Another zinc-conditioned target in BMP signaling is Smad protein phosphorylation. Smad proteins need to be phosphorylated for translocation to the nucleus. Altered intracellular free zinc concentrations led to inhibition of phosphatase activity, subsequently increasing Smad phosphorylation, as demonstrated recently [168]. Smad signaling is highly important for adequate development of Treg and for tolerance induction. If this mechanism is not working accurately, inflammation is triggered. Elevated levels of inflammatory markers, ROS and MMPs are well known to cause tissue injury in numerous tissues. Since EDS is accompanied by disturbed collagen synthesis, and MMPs are one key factor in collagenolysis, MMP malfunction can be considered as a possible explanation for EDS. In this context, zinc signals are crucial because inflammation, as well as MMP hyperfunction, accompanies serum hypozincemia [5,63,66].

Hence, altered intracellular zinc level in EDS might be due to ZIP13 malfunction. For this reason, a possible therapeutic approach could be based on the regulation of the mutant ZIP13 protein stability [300].

### 8.7. Zinc in Inflammatory Disease and Sepsis

ZIP8 influences the innate as well as adaptive immune function during bacterial infection and inflammation and a multitude of other diseases are at least related to ZIP8 malfunction [304,305]. In lung epithelia, ZIP8 is crucial for cyto-protection upon inflammation, mediated by zinc signals [306,307]. Moreover, ZIP8 controls the expression of the pro-inflammatory cytokine IFNγ and its immunological function through the regulation of zinc release from lysosomes [101]. Besides adaptive immunity, ZIP8 also regulates innate immunity through zinc-mediated inhibition of nuclear factor kappa-light-chain-enhancer of activated B cells (NFκB) function in macrophages and monocytes [202].

Besides local inflammation, as described above, systemic inflammation, as observed in sepsis, is frequently observed in the population. In this regard, altered zinc transport mechanisms seem to contribute to the inflammatory effects of sepsis. Most likely, ZIP14, which is inducible by pro-inflammatory stimuli, influences zinc metabolism during sepsis and serves as a target for therapy [63]. Zinc signals act in an anti-inflammatory manner during sepsis by attenuating the pro-inflammatory response, due to cellular zinc uptake by ZIP14. Hence, zinc signals and proper ZIP14 function are essential in pro-inflammatory responses, and zinc deficiency is strongly associated with an elevated risk for exaggerated inflammation and mortality due to sepsis [63,65].

### 8.8. Zinc in Lactation

Some breast-fed full-term infants show apparent clinical showing symptoms similar to Acrodermatitis Enteropathica, such as growth retardation, dermatitis, diarrhea, and alopecia. Studies showed this was the result of zinc deficiency caused by low zinc concentrations in the breast milk [308,309,310]. Zinc in breast milk is essential for the growth and health of neonates, since infantile zinc deficiency is associated with autism spectrum disorders [247]. Genetic analyses indicated that malfunction of ZnT2 was responsible for this condition [309,311,312]. Symptoms can be alleviated by zinc supplementation to the infant, but not to the mother. Interestingly, similar observations were described for mice bearing malfunctioning ZnT4 [313], whereby decreased zinc content in milk was detectable [314]. With time, low zinc-concentrations were lethal for suckling pups. ZnT4 has profound consequences on mammary epithelial cell function and negatively influences nutrition secretion into the milk, and additionally promotes tissue remodeling in the mammary gland during early lactation [315]. Animal studies in mice bearing a malfunction of ZnT4, showed these defects to be associated with decreased Akt expression, and Signal transducer and activator of transcription 5 (STAT5) activation. Moreover, in humans a significantly decreased ZnT5 and ZnT6 mRNA expression was found in fibroblasts and lymphoblasts of mothers secreting zinc-deficient milk [316]. Thus, other ZnT proteins are probably involved in the etiology of this disease. Therefore, future studies are needed to elucidate the entire molecular mechanism of the regulation of ZnT2 expression in mammary epithelial cells to protect breast-fed infants against zinc deficiency and to guarantee proper growth and health of neonates.

### 8.9. Zinc and Diabetes

Diabetes is a chronic disease which damages blood vessels, heart, eyes, kidneys, and nerves. About 340 million people are affected worldwide making diabetes a major cause of morbidity and mortality [317]. Up to ninety percent of patients suffering of diabetes are diagnosed as having type 2 diabetes mellitus (T2DM) and 10–15% type 1 diabetes (T1DM). Whereas T2DM is mainly related on lifestyle, T1DM is an autoimmune disease with an early onset [318]. DM is characterized by loss of glycemic control, disturbed metabolic homeostasis through deterioration of pancreatic β-cell function, and insulin resistance. Besides behavioral factors, hereditary factors also play an important role in determining T2DM risk. The latter include a malfunctioning ZnT8 gene [105]. Thus, ZnT8 transporter is associated with both T1DM and T2DM, respectively [319,320]. Mice that are deficient in ZnT8 have impaired insulin secretion, impaired crystal formation and clear insulin rapidly from the liver [321,322]. Regarding T1DM, ZnT8 autoantibodies are present in 60–80% of patients at the onset of clinical disease [319,323,324]. However, ZnT8 was the last autoantigen uncovered in T1DM, whereas insulin, 65kDa glutamic acid decarboxylase (GAD65) and insulinoma-associated protein 2 (IA-2) where already established long before [318]. Since the prevalence of autoantibodies against ZnT8 has a similar frequency to standard T1DM autoantigens, ZnT8 can be used additionally to detect diabetes-related autoimmunity [325,326]. 

In diabetes, zinc plays a critical role in the formation of insulin crystals in β-cells. Moreover, both insulin and zinc released from β-cells are considered to have crucial paracrine and/or autocrine effects, leading to modulation of various molecular mechanisms [327,328]. In these processes, ZnT8 is essential to guarantee the transport of zinc into secretory vesicles that contain insulin. Those are referred to as insulin granules. The importance of ZnT8 was highlighted by studies investigating ZnT8 knockout mice [322,329,330]. These had significantly decreased zinc contents in β-cells and show only fragmented immature zinc/insulin crystals. Additionally, studies using β-cell-specific, as well as α-cell-specific ZnT8, knockout mice revealed that ZnT8 deficiency contributed to a higher incidence of T2DM through β-cell- and non-β-cell-specific effects, respectively [330,331]. 

Dietary zinc supplementation in mice in vivo was able to attenuate hyperglycemia and hyperinsulinemia, and induced elevated pancreatic zinc concentrations [117], and improved glucose clearance in diabetic rats and mice was observed [123,146]. In line with that, it is well known that zinc-deficient animals are less sensitive to insulin [147]. Oral zinc administration in both mice and humans improves glycemic control in T1DM and T2DM patients, respectively [3]. Since plasma zinc levels in T1DM and T2DM patients were reduced compared to healthy individuals, oral zinc supplementation might be useful as an adjunct therapy to promoting insulin signaling [332,333]. Autoantibody production observed in T1DM might be dampened by zinc supplementation, since especially T cell-dependent antibody production is affected by zinc deficiency [209]. Furthermore, zinc intake may stabilize regulatory T cells as discussed above [72]. This is important in relation to environmental factors such as zinc in drinking water and soils. Although T1DM is clearly related to genetic factors such as HLA-DR3-DQ2 and HLA-DR4-DQ8 [318], the risk of onset of T1DM is associated with low levels of zinc in drinking water and soils [233,334]. Moreover, zinc deficiency triggers the IL-6-induced phosphorylation of STAT3 [145], thereby pathologically enhancing B cell differentiation into antibody-producing plasma cells and autoantibody production. This is due to IL-6 overproduction that is associated with autoantibody production [335].

However, further molecular research needs to be done in this field. ZnT8 may be engaged in the pathogenesis of T2DM in multiple ways and further molecular research is needed to reveal the complete molecular mechanisms responsible for the increased risk to develop DM. 

### 8.10. Zinc and Alzheimer’s Disease

Alzheimer’s disease (AD) was first described in 1906 and accounts nowadays for 60–80% of all dementia cases reported [336]. AD is a chronic neurodegenerative disorder representing the most prevalent form of dementia in modern society. Patients suffering of AD show a progressive cognitive decline. Post-mortem examinations uncovered extracellular deposits, referred as plaques, intracellular deposits, referred as neurofibrillary tangles (NFTs), as well as a significant thinning of cortical grey matter, enlargement of the ventricular spaces, and an overall atrophy of the brain [5]. 

It is well known that a zinc dyshomeostasis is present in AD that might trigger AD-related pathology and symptoms. While the ultimate cause of this zinc imbalance remains to be fully elucidated yet, a dysregulation in various metal storage and transport proteins is likely [337]. 

An imbalanced zinc homeostasis in the hippocampal/parahippocampal gyrus brain area is related to preclinical stage of AD. This is probable due to an increased expression of the zinc transport proteins ZnT4, and ZnT6, and decreased expression of ZnT10 altering zinc concentration and zinc signals in the hippocampal/parahippocampal gyrus [105,338,339]. In addition to that, ZnT1 expression was observed to be reduced in the same brain region individual’s mild cognitive impairment. However, ZnT1 expression was increased in early and late stage AD compared to healthy controls [340]. In accordance to that, zinc-associated activation of tyrosine kinases has been uncovered in patients suffering from AD, leading to misdirection of signaling cascades in neuronal cells of affected patients [112]. Moreover, zinc buffering by MT or calcineurin seems to be altered in AD. Some reports claim changes in MT expression, especially in MT-III [341,342]. However, this is still controversy discussed [343]. Additionally, more recent studies uncovered calcineurin activation to be linked to beta-amyloid (Aβ)-mediated neurodegeneration in AD [344,345]. Analysis of brain tissue uncovered a significant decrease in zinc levels found in AD patients’ neocortex, hippocampus, medial temporal gyrus and thalamus, and superior frontal and parietal gyri [346]. However, these findings are still under discussion [347,348]. Interestingly, high zinc concentrations seem to trigger AD progression, however low zinc concentrations at ratios of 1:0.1 (Aβ/Zn ratio) and 1:0.01 have a protective effect against Aβ toxicity [349,350]. This is in line with findings of Lee et al., showing that ZnT3 KO mice having less vesicular zinc, simultaneously have less cortical and hippocampal plaques [351]. Another explanation of the zinc-driven protective effect against Aβ toxicity is due to zinc binding to Aβ peptide instead of copper. When copper is bound to the Aβ peptide ROS production and cellular damage is triggered [352]. Hence, cellular damage can be reduced by zinc binding to the Aβ peptide. 

In summary, all studies uncover that a disturbed zinc homeostasis may play a role in the pathogenesis of AD. Nevertheless, more research is necessary to uncover potential therapeutic targets to improve patients’ lives.

## 9. Conclusions

**Figure 5:**
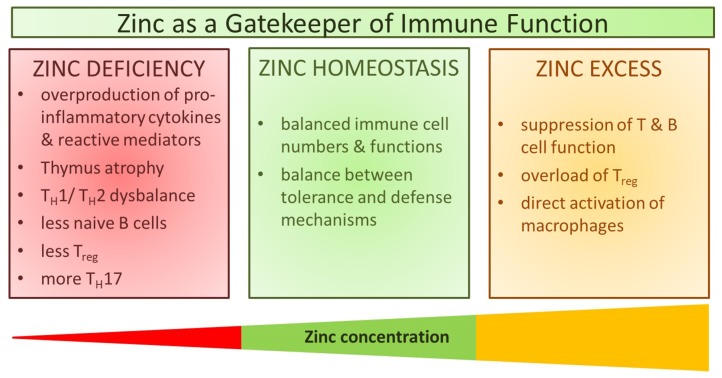
Influence of zinc status on the overall immune function. Adequate zinc homeostasis is essential for a well-functioning immune system. Zinc deficiency as well as zinc excess lead to malfunction of the adaptive and innate immune system, eventually resulting in the development of numerous immune diseases.

Zinc flux, zinc wave, and homeostatic zinc signals control the adequate function of innate as well as adaptive immunity. On the one hand, zinc deficiency causes severe impairment of immune function, comprising the adaptive as well as the innate immune system. On the other hand, high zinc excess provokes an impairment of the immune system comparable to zinc deficiency (see Figure 5). This is why a balanced zinc homeostasis is crucial for either defending against invading pathogens or protecting the human body against an overreactive immune system causing autoimmune diseases, chronic inflammation or allergies. In this regard, zinc can be considered as a gatekeeper of the immune system, since the adequate function of virtually all immune cells is highly zinc-dependent. Knowledge about the molecular mechanisms in zinc-regulated immune reactions is growing and inherited disorders such as Acrodermatitis Enteropathica, as well as nutrition-related immunological malfunctions observed in the elderly, can be treated by appropriate zinc administration. Thus, zinc can be seen as potential therapeutic for clinical use to influence beneficially the well-being of patient’s suffering from immune diseases. However, to completely understand the complex-regulated zinc-triggered immunoreactions, more research will be necessary.
